# Inpactor2: a software based on deep learning to identify and classify LTR-retrotransposons in plant genomes

**DOI:** 10.1093/bib/bbac511

**Published:** 2022-12-10

**Authors:** Simon Orozco-Arias, Luis Humberto Lopez-Murillo, Mariana S Candamil-Cortés, Maradey Arias, Paula A Jaimes, Alexandre Rossi Paschoal, Reinel Tabares-Soto, Gustavo Isaza, Romain Guyot

**Affiliations:** Department of Computer Science, Universidad Autónoma de Manizales, 170001, Caldas, Colombia; Department of Systems and Informatics, Center for Technology Development - Bioprocess and Agro-industry Plant, Universidad de Caldas, 170004, Caldas, Colombia; Department of Computer Science, Universidad Autónoma de Manizales, 170001, Caldas, Colombia; Department of Computer Science, Universidad Autónoma de Manizales, 170001, Caldas, Colombia; Department of Computer Science, Universidad Autónoma de Manizales, 170001, Caldas, Colombia; Department of Computer Science, Universidad Autónoma de Manizales, 170001, Caldas, Colombia; Bioinformatics and Pattern Recognition Group, Department of Computer Science, Federal University of Technology (UTFPR) - Paraná, 80230-901, Paraná, Brazil; Department of Electronics and Automation, Universidad Autónoma de Manizales, 170001, Caldas, Colombia; Department of Systems and Informatics, Center for Technology Development - Bioprocess and Agro-industry Plant, Universidad de Caldas, 170004, Caldas, Colombia; Department of Electronics and Automation, Universidad Autónoma de Manizales, 170001, Caldas, Colombia; Institut de Recherche pour le Développement, CIRAD, Univ. Montpellier, 34000, Montpellier, France

**Keywords:** Inpactor2, LTR-retrotransposons, plant genomes, deep learning, neural networks, detection, classification

## Abstract

LTR-retrotransposons are the most abundant repeat sequences in plant genomes and play an important role in evolution and biodiversity. Their characterization is of great importance to understand their dynamics. However, the identification and classification of these elements remains a challenge today. Moreover, current software can be relatively slow (from hours to days), sometimes involve a lot of manual work and do not reach satisfactory levels in terms of precision and sensitivity. Here we present Inpactor2, an accurate and fast application that creates LTR-retrotransposon reference libraries in a very short time. Inpactor2 takes an assembled genome as input and follows a hybrid approach (deep learning and structure-based) to detect elements, filter partial sequences and finally classify intact sequences into superfamilies and, as very few tools do, into lineages. This tool takes advantage of multi-core and GPU architectures to decrease execution times. Using the rice genome, Inpactor2 showed a run time of 5 minutes (faster than other tools) and has the best accuracy and F1-Score of the tools tested here, also having the second best accuracy and specificity only surpassed by EDTA, but achieving 28% higher sensitivity. For large genomes, Inpactor2 is up to seven times faster than other available bioinformatics tools.

## Introduction

Transposable elements (TEs) are repeated sequences scattered throughout the genome [1]. They have the ability to move from one position in the genome to another, increasing their copy number [2]. The presence of these repeated elements in the genomes is now recognized as a powerful driver of evolution and biodiversity [3], domestication [3, 4] and variations in genome size [5]. According to their transposition mechanism, they are first divided into two classes: Class I or ‘retrotransposons’ and Class II or ‘transposons’. They are further sub-classified hierarchically into subclasses, orders, superfamilies, lineages and families [6, 7]. Long terminal repeat (LTR) retrotransposon (or LTR-RT), an order of Class I elements [8] comprise the most abundant elements in plant genomes [9, 10]. LTR-RTs move via a copy-and-paste mechanism, using an RNA intermediate that is reverse transcribed into cDNA and introduced into the genome by a integrase coded by the full LTR-RT copies [6, 11]. They are sub-classified into two superfamilies found in a large number of eukaryotes, namely Ty1/*Copia* and Ty3/*Gypsy* [12], which differ structurally by the order of the internal coding domains [13]. They can be further sub-classified into lineages according to similarities of their domains [14].

Machine Learning (ML) techniques have been used to solve several genomic and evolutionary problems of biological systems [15]. ML is defined as the use of calibrated computational algorithms based on previous experiences through statistical inference on the data to make predictions in classification and regression problems [16, 17]. Its main function is to tune the parameters needed to optimize performance on training data and subsequent input data [18, 19].

Some researches have used ML models for TE identification, for example, Orozco-Arias *et al.* (2019) [13] reviewed the use of ML for the analysis of TEs and Loureiro *et al.* (2013) [20], Nakano *et al.* (2018) [21] and Panta *et al.* (2021) [22] studied how to improve the accuracy and performance of TE classification. Recently, pre-processing techniques and coding schemes that enable deep classification of TEs have been studied [23, 24]. Besides, software such as RED [25], PASTEC [26], TEClass [27] and TransposonUltimate [28] apply ML techniques such as Support Vector Machines, Random Forests, Hidden Markov Models, K-nearest Neighbors, Neural Networks (NN) and graphical models due to feature extraction, process automation and faster algorithm executions.

To identify and classify TEs, bioinformaticians have developed many tools, techniques and methods, including structure-based, homology-based, *de novo* and comparative genomics [29–31]. The most accurate tools combine several methods to improve their results, such as LTR-FINDER [32], EDTA [33] and Inpactor version 1 [34]. Nevertheless, this makes the tools slower in execution especially in large genomes, taking hours or even days, which, together with high variability and redundancy of TEs [35], makes it unfeasible to process the large amount of genomic data that are released every day [36].

In recent years, several datasets consisting of thousands of TEs from various species have been created and published, such as Repbase [37], RepetDB [38], PGSB Plants DB [39] and InpactorDB [40]. These datasets constitute valuable resources for improving tasks such as TE detection and classification and have motivated the proposal and evaluation of ML techniques to obtain substantial results in terms of accuracy and speed in executing these tasks [20, 31].

Although good results are obtained using current ML techniques, such as ordinary NN, recent advances have shown that Deep Neural Networks (DNNs) can achieve better results, in which non-parametric models based on NN are implemented to adapt associations between input and output data [41, 42]. In this field, several DNN architectures have been published, such as the Fully Connected Neural Network (FNN) by [21], the Convolutional Neural Network (CNN) with 2D representation of sequences by [42, 43] and the 1D CNN for classifying TEs into superfamilies by [44]. However, none of these NNs have been integrated into a single software that automatically detects and classifies TEs in relatively short times.

Additionally none of the DL approaches or current tools (with the exception of TEsorter [45]) perform the task of classifying LTR-RTs at the lineage/family level to obtain accurate results. Here, we present Inpactor2, a novel method and tool based on four NN that detect and classify LTR-retrotransposons automatically. This tool was executed in plant genomes up to $2.2$ Gb and obtains results up to seven times faster than state-of-the-art tools decreasing the execution time from more than 20 hours to less than three. Moreover, using the rice genome *Oryza sativa*, Inpactor2 gets the best performance in accuracy ($96.1\%$) and F1-Score ($91.9\%$) and the second best in specificity (97.7%), precision (92.7%) and in false discovery rate (FDR) (7%) of the tools tested here. Inpactor2 is freely available at https://github.com/simonorozcoarias/Inpactor2. Additional information about NNs architectures used, and their hyper-parameters can be found at https://github.com/simonorozcoarias/Inpactor2/tree/main/NN_architectures.

## Materials and methods

### Genomic datasets used for training of NNs

Inpactor2 was designed to perform three main tasks in the analysis of LTR-RTs, detection, filtering and classification. Each task is done by a different NN. Thus, detection is done by a CNN named Inpactor2_Detect, filtering by an FNN called Inpactor2_Filter and classification by another FNN named Inpactor2_Class. Thus, each architecture needed different datasets to be trained. For example, Inpactor2_Detect was trained on a dataset consisting of $70\,000$ sequences of $50\,000$ bases in length. To create the target sequences (those regions with LTR-RTs in them), we randomly obtained an LTR-retrotransposon from InpactorDB [40] and placed it at a random position. Then, the remaining one was filled with sequences corresponding to other genomic features obtained from [24] (DOI 10.5281/zenodo.4543904). For negative sequences (those without LTR-RTs within), sequences of genomic features other than LTR-retrotransposons were again obtained from [24] (DOI: 10.5281/zenodo.4543904). This dataset was composed of $35\,000$ sequences with LTR-RTs (target sequences) and $35\,000$ sequences without these elements (negative sequences).

On the other hand, Inpactor2_Filter was trained using a dataset with binary labels, where zero corresponded to intact sequences and one corresponded to non-intact. All the sequences presented in InpactorDB (67 241 elements) were used as intact elements, while non-intact sequences were taken from those filtered by [40] and corresponded to (a) sequences with a LTR-RT inserted into another (from different superfamilies or lineages), (b) sequences of lengths shorter or larger than those reported in the Gypsy Database [46] (with a tolerance of $20\%$) and (c) sequences with TEs class II inserted into LTR-RTs. In total, this dataset contained $105\,657$ sequences. As features, Inpactor2_Filter used *k*-mer frequencies using $1\leq k\leq 6$.

Finally, Inpactor2_Class was trained using InpactorDB after *k*-mer frequencies extraction in the same way as Inpactor2_Filter. This dataset comprised $67\,241$ LTR-RTs classified at the lineage/family levels. *k*-mer frequencies were estimated in each sequence by calculating all possible *k*-mers with a maximum length of six nucleotides and later counting the number of occurrences [23, 24].

### Benchmarking the performance of Inpactor2

The reference *O. sativa* genome [47–51], was selected for testing the software due to its high-quality assembly, small genome size (389 Mb) and quality of its genes and TEs annotations. The *O. sativa* genome was identical to the one used by [33] to compare the results with benchmarking tools in this study. This work used the standard library v6.9.5 created by [33] based on the *O. sativa* L. ssp. *japonica* cv. ‘Nipponbare’ v. MSU7 genome and RepeatMasker v4.0.8 [52] with the following parameters ‘-pa 36 -q -no_is -norna -nolow -div 40 -cutoff 225’.

Additionally, six different plant genomes (Table 1) were used to test the execution times of Inpactor2 by assessing different genome sizes and TE compositions. The genomes were downloaded from NCBI and analyzed with Inpactor2 using the following parameters (-m 15000 -n 1000, -i no, -d no, -C 1, -c yes -a no), as suggested in [53]. Finally, EDTA was run with the same genomes to compare its execution times with Inpactor2. EDTA was executed using EDTA_raw.py script, –type ltr, and the other parameters by default.

**Table 1:** Plant genomes used in the execution time tests

Species	Assembly size (Mb)	Accession number
*Arabidopsis thaliana*	121.2	GCF_000001735.4
*Oryza sativa*	379.1	GCF_001433935.1
*Coffea canephora*	579.4	GCA_900059795
*Solanum lycopersicum*	839.1	GCF_000188115.4
*Coffea arabica*	1126.4	GCF_003713225.1
*Zea mays*	2252.8	GCF_902167145.1

Libraries of LTR-RTs of the species shown in Table 1 were then created using Inpactor2 (with and without filtering with the -c flag) and EDTA. In addition, two species that were not contained in the training data were used, such as *Coffea humblotiana* [54] and *Gardenia jasminoides* [55]. These libraries were then annotated using repeatMasker and compared with the proportion of genomes corresponding to LTR-RTs according to the papers where the genomes were reported. A workstation with AMD Ryzen Threadripper 3970X 32-Core Processor, 128 Gb in RAM memory and a GPU Nvidia RTX 2080 super was used to perform all the experiments.

**Figure 1:**
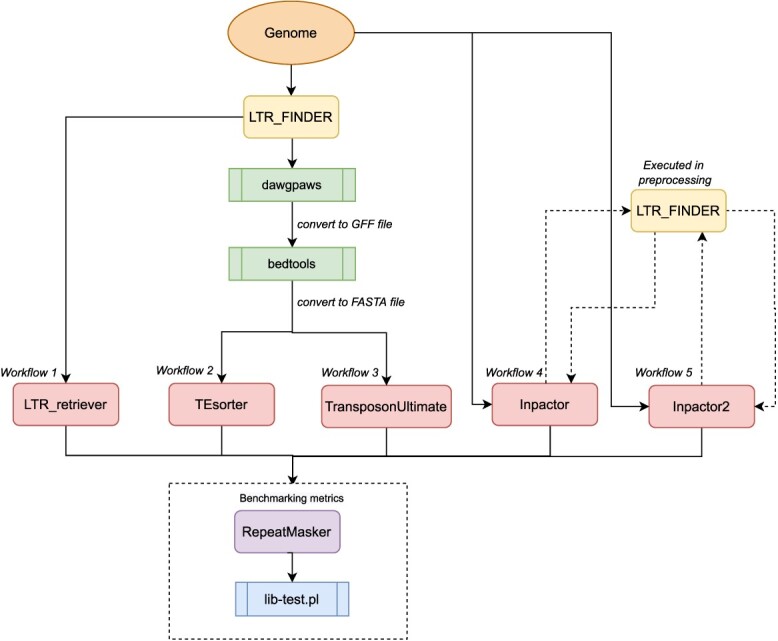
Workflow of the benchmarking process. Firstly, LTR_FINDER was executed as detector software for each workflow. Then, because TEsorter and TransposonUltimate use as input a FASTA-format file, dragpaws v.3.0 (http://dawgpaws.sourceforge.net/man.html) was executed to convert the LTR_FINDER’s output to GFF and bedtools v.2.29.2 (https://bedtools.readthedocs.io/en/latest/index.html) was utilized to convert it to FASTA. Both, Inpactor version 1 and Inpactor2 used LTR_FINDER in their own workflow. LTR_Retriever was the unique tool that uses the raw LTR_FINDER’s output. Finally, using the library created by each workflow, RepeatMasker was executed and the perl script named lib-test.pl [33] was utilized to get the performance metrics.

**Figure 2:**
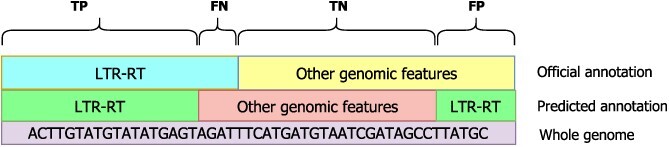
Metrics evaluated for each LTR-RT classifier. Based on [33]. TP corresponds to true positive, FN to false negative, TN to true negative and FP to false positive.

To evaluate the performance of Inpactor2 compared with other software, a similar methodology to the one proposed in [33] was followed. First, Inpactor v.1.0 [34], TEsorter v.1.3 [45], Transposon Ultimate v.1.0 [28], LTR_retriever v.2.9 [56] and LTRharvest [57] were selected for benchmarking given their methodologies for classifying LTR-RTs to the superfamily level. A workflow was established for each software, initially using LTR_FINDER v.1.0.7 as the LTR-RTs detector. Then, the *O. sativa* genome was annotated with RepeatMasker and performance metrics were extracted for each workflow. The metrics evaluated were: accuracy, precision, specificity, sensitivity, FDR and F1-score. Figure 1 shows the schematic representation of the benchmarking metrics. In this study, TP, FN, TN and FP are the number of nucleotides belonging to each category (Figure 2).

The script called ‘lib-test.pl’, included in the EDTA toolkit [33], was used to extract the six metrics. Since this study only focused on the LTR-RT category, so the script was executed using the -cat ltr parameter to perform the comparative evaluation.

## Results

### General architecture of Inpactor2

**Figure 3:**
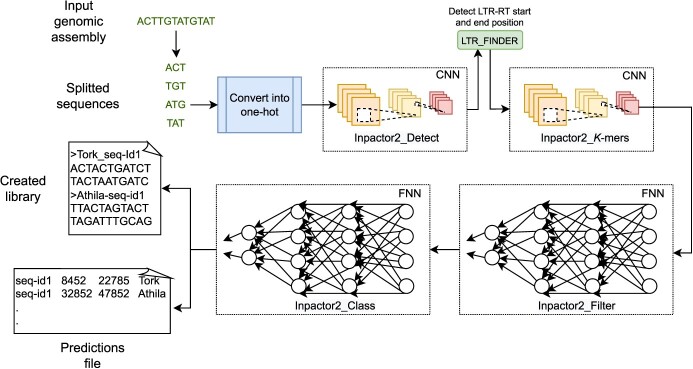
General schematic representation of the Inpactor2 workflow. The tool’s core is composed by four NN. Inpactor2 receives as input the genomic assembly, then it splits the sequence into non-overlapping sections of 50 kb length. As outputs, Inpactor2 creates a library in FASTA format with the detected and classified LTR-RTs and a tabular file with the predictions made by the three NNs. A detailed graphical schema of the Inpactor2 workflow and each section can be found at Figure S1.

Inpactor2 is composed of four NNs, each one performs one function in the pipeline. First, Inpactor2 receives a genome assembly in FASTA format, then each sequence in the input file is cut into 50 kb sections, without overlapping. Each section is converted into a 2D-representation using one-hot encoding. This coding scheme generates a $5 x n$ matrix of $ones$ and $zeros$, where each row corresponds to the possible nucleotides (A, C, T, G or N) and the columns represent the bases present in the sequence of length $n$. This coding puts a $1$ in the position of the row that corresponds to the base of the sequence. For an example see [43] and the section ‘Inpactor2_*K*-mers network definition’. Next, a CNN called Inpactor2_Detect is used to predict which section contains LTR-RTs and these segments are retained for further analysis. This network has the only function of retaining those sections of interest in the genome and eliminating those that do not contain LTR-RTs (according to the predictions). In this way, the execution time and memory required for the following steps is optimized. Next, LTR_FINDER is run on the sections that were predicted to contain LTR-RTs inside by Inpactor2_Detect to search for the start and end positions of the detected LTR-RTs. This step is executed in parallel to reduce the execution time. After, a CNN called ‘Inpactor2_*K*-mers’ is used to count *k*-mer frequencies in the extracted LTR-retrotransposons. This CNN is intended to extract features required by the following NNs of the pipeline in a time-efficient way (See ‘Inpactor2 *K*-mers network definition’ section). Intact and potentially complete LTR-retrotransposons are filtered and retained based on the *k*-mer frequencies and a FNN called ‘Inpactor2_Filter’. Finally, a FNN named ‘Inpactor2_Class’ is used to classify the elements into lineages. Figure 3 shows a schematic of the general structure of Inpactor2.

Inpactor2 can be executed using several cycles (from one to five) of analysis, where each cycle splits differently the input sequences in order to predict the elements that remain split in any of the partitions. This behavior is controlled with the parameter -C (upper case), and its default value is 1. Additionally, Inpactor2 can be performed using different structural parameters to filter LTR-RTs, such as minimum and maximum LTR-RT length, LTR domains starting with TG and finishing with CA, and target site duplication (TSD) before and after the element. These parameters are given with the flags -M, -m, -i and -d, where their default values are 2000, 28000, yes and yes, respectively.

**Figure 4:**
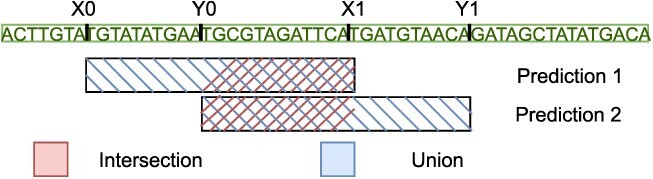
IOU operation between two overlapping LTR-RT predictions along a chromosome sequence.

After finishing all cycles, Inpactor2 removes the predictions that are non-maximal, following the same methodology of the Yolo architecture [58]. An analogous concept is adapted from there, namely the intersection over union (IOU) operation (also named as Jaccard index). Here, the IOU operation between two LTR-RT predictions is defined as the number of nucleotides overlapping in both predictions (intersection) over the total number of nucleotides covered in the chromosome by both predictions (union) (Figure 4). If two predictions have an IOU score > 0.6, then, Inpactor2 only keeps the one with best prediction score. This score is calculated as the average of the probabilities obtained by each NN (Inpactor2_Detect, Inpactor2_Filter and Inpactor2_Class). These probabilities can be consulted in the Inpactor2’s output file named ‘Inpactor2_predictions.tab’.

The IOU score is defined in the Equation 1

(1) \begin{align*}& IOU = \frac{max(0, min(Y1, X1) - max(Y0, X0))}{max(Y1, X1)-min(Y0,X0)}, \end{align*}

where X0 and Y0 are the beginning positions of predictions 1 and 2, and X1 and Y1 are the ending positions of predictions 1 and 2, respectively (Figure 4).

### Inpactor2_Detect network definition

To reduce the execution time, Inpactor2 first splits the input sequences into 50 kb sections and predicts which section may contain LTR-retrotransposons. To do this, a CNN was designed based on the TERL architecture [43]. Inpactor2_Detect is composed of three convolutional layers, each one followed by a max pooling layer. After the convolutional layers, two fully connected layers are used to obtain the predictions of 1000 and 500 neurons, respectively (Figure S2). The activation function in all layers was ReLu, whereas SGD was used as optimizer and binary cross-entropy was used as loss function. The network was trained using 100 epochs and a batch size of 64. The Inpactor2_Detect performance metrics can be consulted in Table S1.

### Inpactor2_*K*-mers network definition

The software proposed herein performs pre-processing of the information stored in the extracted LTR-RT sequences. This algorithmic treatment includes the computation of *k*-mer frequencies, a principal component analysis and scaling. Although there are bioinformatic algorithms to compute the *k*-mer frequencies, a CNN is proposed in this paper for counting 1-mers to 6-mers in DNA sequences. However, to accomplish this task, the DNA sequence must be first transformed to a digital encoding. According to [43], a suitable representation of the DNA is the one-hot encoding, so a modification of this is used henceforth. The one-hot encoding used here only has five rows: A (adenine), C (cytosine), G (guanine), T (thymine) and N (unidentified nucleotide).

**Figure 5:**
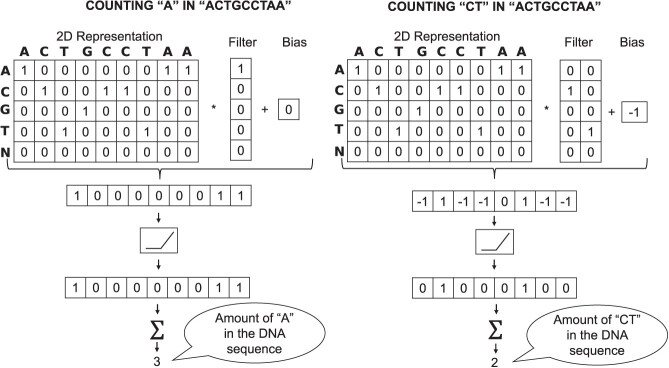
Convolutions used for *k*-mer frequencies computations.

To understand how a CNN can be used to compute *k*-mer frequencies, two examples are considered in Figure 5. The first one is computing the amount of ‘A’ in a DNA sequence ‘ACTGCCTAA’, whereas the second example is computing the amount of ‘CT’ in the same DNA sequence. According to the Figure 5, the weights and biases can be set manually to compute the frequency of any *k*-mer. For example, the amount of a specific *k*-mer in a DNA sequence can be computed if the weight matrix is set equal to the 2D representation of that *k*-mer and the bias is set to $1-k$.

Considering that a filter can have more than one dimension, these can be used to compute all the *k*-mer frequencies at once, accelerating the counting time compared with the conventional counting method (Table S2). The final CNN architecture is illustrated in Figure S3.

Inpactor2_*K*-mers takes mathematical representations of DNA sequences with dimensions of 5x50000x1 and extracts the frequencies of 1–6-mers in lexicographic order. Therefore, the frequencies of $4^1+4^2+4^3+4^4+4^5+4^6 = 5460$*k*-mers can be computed by the developed CNN.

### Inpactor2_Filter network definition

To obtain intact LTR-retrotransposons, ML-based experiments were developed. For this, sequences from InpactorDB [40] named class 0 (intact elements) are used, whereas the elements that were removed in each of the filters proposed in the same study were taken as non-intact sequences named class 1.

Using the produced dataset, a NN based on the FNN proposed in [21] was trained and tuned. The developed architecture, called Inpactor2_Filter, is shown in Figure S4. For each of the layers, a dropout of 0.5 and a ReLu activation function were utilized using BatchNormalization with a momentum of 0.99. For the prediction layer, a softmax activation function was used. Adam optimization algorithm was applied to find a suitable configuration of the NN and categorical Cross-entropy was used as the loss function. Furthermore, regularization l1 was added to kernel with value of 0.0001 and l2 to bias with a value of 0.01. These regularizers were applied to the three hidden layers. For the training stage, 200 epochs were used and the batch size was set to 128.

Performance metrics are shown in Table S1 to evaluate the correct generalization of the model.

### Inpactor2_Class network definition

Following detection and filtration, LTR-RTs were classified into lineages. A FNN was designed with three hidden layers, each one with 200 neurons. The Inpactor2_Class structure is similar to Inpactor2_Filter (Figure S4), but with 13 output neurons to implement the multi-class classification. This network was trained for 200 epochs with 128 as batch size, using InpactorDB [40] sequences converted to *k*-mer frequencies. Inpactor2_Class obtained a performance of 0.98 in accuracy, precision, recall and F1 score (Table S1).

### Inpactor2_utils

In addition to the main component of Inpactor2, an extra script that contains utilities for the LTR-RT analysis was released, such as deletions of characters different from nucleotides (A, C, T, G or N), calculation of *k*-mer frequencies with $1\leq k\leq 6$ using Inpactor2_*K*-mers architecture, re-training Inpactor2_Class to specialize the NN for a specific group of species, among others. Inpactor2_utils is available in the same repository than the main script.

### Benchmarking results

**Figure 6:**
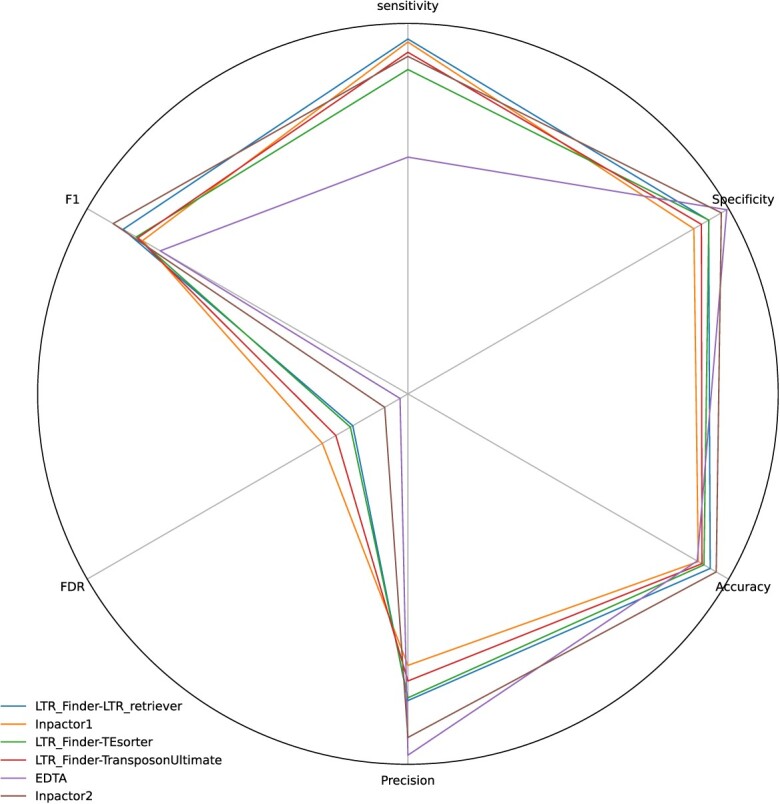
Benchmarking of LTR-RT classifier tools. All the results obtained by each tool in each of the metrics can be consulted in Table S3.

Following the proposed methodology, an evaluation of sensitivity, accuracy, precision, specificity, FDR and F1-score metrics, as well as false positive, false negative, true positive and true negative rates was performed for the selected software (Figure 6 and Table S3). An accuracy of 96.1% was obtained for Inpactor2, representing the highest value for that metric, as well as an F1-score of 91.9%. However, for specificity, precision and FDR, Inpactor2 obtained the second best values with 97.7%, 92.7% and 7%, respectively only surpassed by EDTA. Nevertheless, EDTA showed the lowest value of sensitivity, whereas Inpactor2 obtained 28% more in this metric.

### Execution time comparisons

For each workflow, the execution time was counted to determine which of the selected software was the fastest for the detection and classification of complete LTR-RTs (Figure S5). The best execution time is given by Inpactor2, with 5.47 minutes (in the *O. sativa* genome), constituting the fastest software for the creation of complete LTR-RT libraries.

Additional execution time tests were performed using different genomes sizes ranging from 121.2 Mb for *Arabidopsis thaliana* to 2,252.8 Mb for *Zea mays*. In these tests, only EDTA and Inpactor2 were executed. Table S4 shows that Inpactor2 is faster than EDTA, specially with larger genomes (i. e. up to seven times faster for the biggest genome tested, *Z. mays*).

The next step consisted in creating libraries of the genomes shown in Table 1 and of the species of *C. humblotiana* and *G. jasminoides* using three different flows, Inpactor2 doing the curation of the sequences (with the Inpactor2_Filter network), Inpactor2 without process of curating and EDTA. At the end, the libraries were used to annotate the LTR-RTs and compare the proportions of each genome that correspond to each library against what was reported in the original papers. Figure S6 shows a large variability between the different streams with respect to the original annotation. This could be because there is no standard annotation process for TEs and therefore different investigations use different characteristics to consider whether or not a sequence should be in the annotation library. When specifically comparing EDTA and Inpactor2 (Figure S7) with filtering turned on (at the library level) we found that some of the LTR-RTs contained in the Inpactor2 library were the representation (in average) of up to 41 models in the EDTA library (Figure S7I). This observation is in line with that reported by [59] in other organisms such as *Drosophila melanogaster*. However, it is interesting that although EDTA appears to create many more models than Inpactor2, at the annotation level (Figure S6) the difference is much less evident.

## Discussion

TEs represent the main component of plant genomes. They are sources of mutations and polymorphisms responsible of the phenotype variability in numerous crop species. For instance, they influence grape color [60], pigment instability in corn kernels (from completely yellow to spotted kernels to completely purple), tomato shape and size [61], potato skin color [62], orange color and flavor [63] and the presence or absence of fuzzless in peach skin [64]. In addition to phenotypic implications, others researches have shown that these elements have key roles in other aspects such as contributing to intra-species diversity [65], influencing gene expression [66, 67] and potentially adaptation to climate changes [68]. With the improvement of genomic sequencing techniques, a large amount of plant genomes is now available in public sequence databases. In addition the sequencing of large and complex genomes, containing a high amount of TEs, is becoming possible at moderate costs. These genomes make possible the analysis of TEs with the accessibility of a variety of annotation pipelines using different strategies, such as structure-based, homology-based, *de novo* and comparative genomics-based [13, 20, 29–31] and, lately, ML-based (or alignment-free) [21, 22, 24, 28, 31, 40, 42–44].

ML-based (or alignment-free) strategies are clearly well adapted for analyzing numerous and large genome size, but require a high-quality and representative training dataset to be applied with reliability on different species; otherwise, they will not make good predictions on species far from those contained in the training dataset. However, at the computational level, the analysis of genomic datasets (and more particularly TEs) is particularly complicated with ML-based approaches due to the size, the relatively unstructured format and the complex visualization of the data. Moreover, since each nucleotide is represented by a letter (categorical data type), a transformation (or a feature extraction) is required to process the data. *K*-mers have proven to be crucial features [24] to implement ML models [69, 70] and NN architectures [21, 44]. Nevertheless, counting *k*-mer frequencies requires computational time and displays issues with space consumption and scalability [71]. This fact can negatively affect the entire process of the software, specially in genomes with many LTR-RTs, becoming a bottle neck in NN-based tools. Thus, by taking advantage of GPUs, Inpactor2_*K*-mers offers the possibility to compute *k*-mer frequencies faster than using CPU-based and conventional methods (Table S2) overcoming this problem. By using filters from a convolutional network, each of the required 5460 *k*-mer frequencies can be calculated from matrix operations, which libraries such as Tensorflow can execute on CPU or GPU without user input. In addition, by simultaneously counting all *k*-mers and using the batch size parameter (depending on the amount of memory available on the GPU device) the computation time can be reduced considerably.

Inpactor2 was designed with the objective to produce highly accurate libraries of complete LTR-RT sequences in plant genomes and to optionally annotate them in genome assemblies. Inpactor2 is a hybrid tool that basically combines four NNs for detection, providing sensitivity and speed of execution, with a structure-based software (LTR_FINDER) to extract potential candidates and finally with a homology-based tool (RepeatMasker) to annotate all fragments in assemblies.

Further Results obtained by Inpactor2_Detect and LTR_FINDER at the full region level (Figure S8 and Table S5) using the genomes of *A. thaliana* and *O. sativa* allowed us to better understand how the hybrid approach processes the genomic data. We were able to conclude that only 1.3% in *A. thaliana* and 3.7% in *O. sativa* of all the regions into which the genome was divided contained elements according to LTR_FINDER, but were not detected by Inpactor2_Detect (column ‘LTR_FINDER only’). On the other hand, we found that 45.8% in *A. thaliana* and 25% in *O. sativa* of all regions did not contain LTR-RTs according to both approaches (column ‘Both negative’). This allows the software to not run LTR_FINDER (which is much slower than Inpactor2_Detect) only on these portions of regions that were found by Inpactor2_Detect.

The methodology implemented in Inpactor2 (in Inpactor2_Detect) allows increasing the sensitivity of detection compared with other software, such as EDTA and LTR_FINDER. The FDR remains very low due to the filtration performed by Inpactor2_Filter, which retains only intact or complete sequences. This curation process is a crucial task to achieve good quality masking and annotation because if element fragments (for example soloLTRs) are present in the library, which come from intact (or complete) LTR-RTs that are also in the library, the annotation could show an over-estimation of the element contribution. Since it would take into account both the whole element and its fragments [72]. Finally, Inpactor2_Class assigns a lineage to each predicted complete element. This deep level of classification is very rare in classification tools, which typically reach only the superfamily level (i.e. *Copia* an *Gypsy*), such as in EDTA, TransposonUltimate and LTR_retriever.

Inpactor2 is composed of four NNs, each one designed for a specific task and trained with a different and specially designed dataset. This approach allows each NN to have a relevant performance. For example, Inpactor2_Detect has an accuracy, precision, recall, and F1-score of 97%, Inpactor2_Filter has 91% in the same metrics and Inpactor2_Class has 98% in the metrics mentioned above (Table S1). Altogether, these NNs (along with Inpactor2_*K*-mers) integrate a pipeline that is up to seven times faster than popular tools such as EDTA (Table S4), while maintaining the performance showed by software such as LTR_FINDER, LTR_retriever and Inpactor version 1. Inpactor2 was designed to be easy to install (within an Anaconda environment) and to execute. Also, Inpactor2_utils.py provides more utilities to facilitate the implementation of the entire pipeline. Finally, Inpactor2 can be easily re-trained with data from specific plant genera or orders to create specialized models, thus increasing the performance of the tool and accelerating studies that simultaneously cover many species.

Together our data suggest that Inpactor2 can be a reliable and rapid tool to build libraries and annotate LTR-RTs in plant genomes and to analyze these sequences in a comprehensive and reproducible manner. As an a example, we searched within the *O. sativa* genome for five well-known LTR-RTs and all of them were found in the Inpactor2’s library with a BLASTn identity higher than 90% (Table S6). However, it should be kept in mind that the performance of Inpactor2 depends on two essential factors: the quality and representativity of the dataset used for training Inpactor2 and of course the quality and the contiguity of the genome assemblies analyzed. In the near future, more large-scale LTR-RT analysis (such as the LTR-RT analysis of 300 plant species released by [73]) and high-quality genome assemblies will contribute to generate more datasets that can be used for re-training Inpactor2, increasing again its performance. Also as future work, it is proposed to generate and include datasets of other groups of organisms such as animals and fungi, as well as to include other TE taxa following the same methodology shown in InpactorDB.

## Conclusion

Inpactor2 is a four-NN-based tool that reduce the execution time of complete LTR-retrotransposon library creation from plant genomes. This software detects, and classifies complete LTR-retrotransposons in few minutes (up to 26 minutes for *C. arabica*, 1.1 Gb), speeding up the computational time up to 7.1 times compared with EDTA (using *Z. mays* genome) and constituting the fastest tool tested in this study. Our benchmarking suggests that Inpactor2 is more sensitive, accurate and has a higher F1-Score than EDTA. Also, it has a higher specificity and precision and lower FDR than LTR_FINDER, Inpactor version 1, TEsorter and TransposonUltimate.

**Key Points**

- The hybrid approach used by Inpactor2 allows the creation of quality LTR-retrotransposon libraries, maintaining a high level of precision, accuracy and sensitivity and keeping a low number of false positives.
- Inpactor2 can be run using CPUs + GPUs, speeding up the execution time up to 7 times, being the fastest software in the creation of libraries of the tested software. This allows to analyze more genomes in less time, being useful for large scale analysis.
- Although other software is capable of identifying and classifying TEs in genomes, such as REPET, Inpactor2 is the first available tool that integrates the process of detection, curation and classification at the lineage level in a single software package and in a reasonable computation time. It is also easy to install and use, eliminating the need for manual operations.
- Inpactor2 is the first NN-based tool to detect LTR-retrotransposons *de novo*. It can be installed in an anaconda environment and can be run in a single Python command.

## Data availability statement

The data underlying this article are available in the article and in its online supplementary material.

## Author contributions statement

R.G., G.I. conceived the experiment(s); S.O.-A., L.H.L.-M., M.S.C.-C., M.A. and P.A.J. conducted the experiment(s); S.O.-A. and R.T.-S. analyzed the results; S.O.-A., L.H.L.-M., M.S.C.-C., M.A., P.A.J., A.R.P., R.T.-S., R.G. and G.I. wrote and reviewed the manuscript.

## Supplementary Material

Supplementary_Materials_RG_bbac511

## Author Biographies

**Simon Orozco-Arias** received the B.S. degree in System and Computing Engineering and the Ph.D. Degree in Engineering (Bioinformatics) from the Universidad de Caldas, Colombia, in 2016 and 2022, respectively. He is currently working as professor and researcher with the Universidad Autónoma de Manizales, developing projects on bioinformatics, supercomputing, and data analysis. He has contributed to the solution of biological problems, especially, related to plant DNA. His current research interest includes the application of machine learning techniques to automatically extract knowledge from DNA data.

**Luis Humberto Lopez-Murillo** was graduated in chemical engineering in 2018 and candidate to M.Sc chemical engineering from Universidad Nacional de Colombia. Scientific researcher at Universidad Autónoma de Manizales. Research fields: (1) Deep learning applied to bioinformatics and (2) Process system engineering applied to membrane technologies. Member of research groups: (1) Ingeniería de software at Universidad Autónoma de Manizales (2) Grupo de Investigación en Aplicación de Nuevas Tecnologías (GIANT) at Universidad Nacional de Colombia.

**Mariana S. Candamil-Cortés** is a Biomedical Engineer from Universidad Autónoma de Manizales. Her research interests are bioinformatics, computational biology and data science.

**Maradey Arias** is a biomedical engineer from the Universidad Autónoma de Manizales (Colombia), and during her association with the Bioinformatics and Artificial Intelligence research group, she worked on the identification and classification of transposable elements in plants, using bioinformatics tools and machine learning techniques.

**Paula A. Jaimes** is a biomedical engineer from the Universidad Autónoma de Manizales, Colombia, belonging to the Bioinformatics and Artificial Intelligence research group since 2019. She has been working in genomics field, focused on the identification and classification of transposable elements in plants. Her current research interest includes the understanding of the DNA data through bioinformatic tools and machine learning techniques.

**Alexandre Rossi Paschoal** is an associate professor at the Dept of Computer Science - Federal University of Technology – Parana, UTFPR, Brazil. He received his Ph.D. in Bioinformatics at the University of São Paulo in 2012. His research includes data science, bioinformatics, and pattern recognition approaches to model and understand biological data.

**Reinel Tabares-Soto** received the B.S. degree in electronic engineer from the Universidad Nacional de Colombia, in 2009, the B.S. degree in systems and computer engineer from the Universidad de Caldas, Colombia, in 2016, the M.S. degree in engineering from the Universidad Nacional de Colombia, in 2017, and the Ph.D. degree in computer science from the Universidad Autónoma de Manizales, Colombia, in 2021. He has been a Professor and a Researcher with the Department of Electronics and Automation, Universidad Autónoma de Manizales, since 2014. He has been also a data science consultant for different public and private companies in Colombia since 2017. He is currently holding a Postdoctoral position at the Adolfo Ibañez University in the framework of the ethical and responsible algorithms project. His main research interests include steganalysis, machine learning, deep learning, bioinformatics, and high-performance computing.

**Gustavo Isaza** is a Ph.D. in Software Engineer from Universidad Pontificia de Salamanca, Spain, 2010, received a B.S. degree in system and computing engineering from Universidad Autónoma de Manizales, Colombia, in 1997. He obtained a postgraduate degree in networking software development from Universidad de los Andes, Colombia, in 1998. He is full Professor at Universidad de Caldas, Senior Researcher from MinCiencias (Ministry of Science, Technology and Innovation) of Colombia and a member of the GITIR Research Group and CDT Bioprocess and Agro-industry Plant researcher. He has published over 70 papers and conferences related to machine learning in cybersecurity, bioinformatics, AI in videogames, and distributed computing.

**Romain Guyot** is a research director at the Institut de Recherche pour le Développement. He has extensive experience in transposable elements genomics and bioinformatics.
