# Supplementary material for: Inpactor2: a software based on deep learning to identify and classify LTR-retrotransposons in plant genomes
Source: Brief Bioinform. 2022 Dec 10;24(1):bbac511. doi: 10.1093/bib/bbac511 (PMC9851300; doi:10.1093/bib/bbac511)
Supplement: Supplementary_Materials_RG_bbac511 [file supplementary_materials_rg_bbac511.docx]

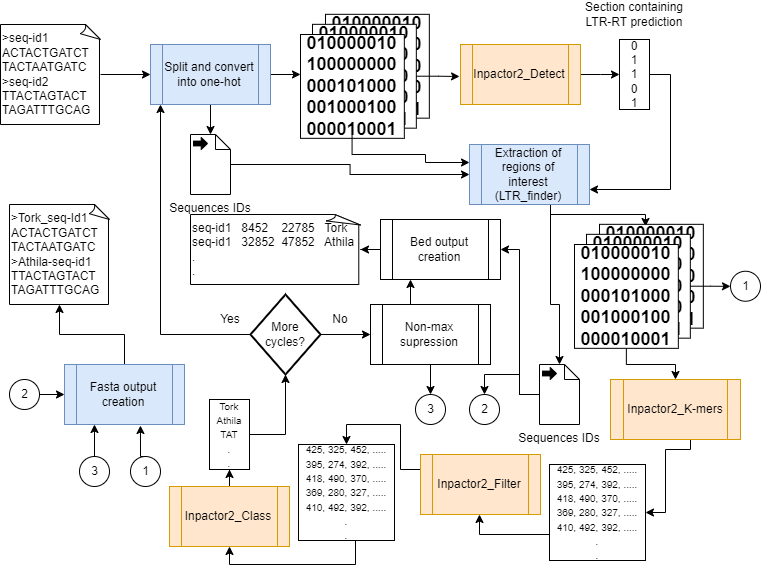


**Figure S1**. A detailed graphic schema of Inpactor2. The entire process start with a genomic assembly in Fasta format. Then, these sequences are split in 50 kb sections and converted into one-hot representations (2D matrices of zeros and ones). Those matrices are entered into the Inpactor2_Detect network to predict which sections contains LTR-RTs. Its output is a 1D-array of zeros and ones. Using Inpactor2_Detect’s predictions and the 2D one-hot matrices, another process is run to extract only the sequences of the LTR-RTs found by LTR_finder (Xu et al., 2007). Following, Inpactor2_K-mers counts the *k-mer* frequencies (using 1<=k<=6) that will be used by Inpactor2_filter. That network removes sequences that seems to be non-intact. Finally, Inpactor2_Class network classify the LTR-RTs into lineages. The blue boxes represent process that run in multiple cores, light orange boxes are neural networks (they can be run in GPUs if available), and white boxes are auxiliary functions.


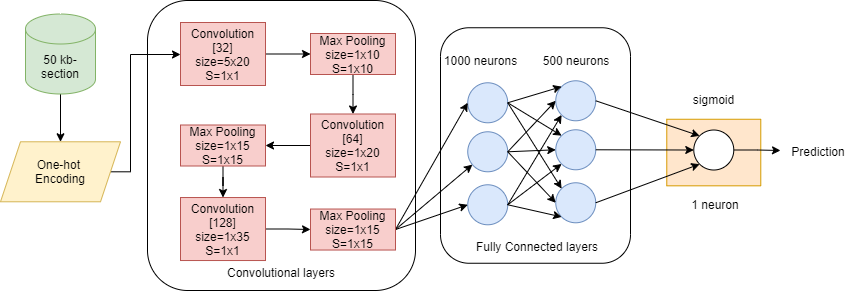
**Figure S2.** Inpactor2_Detect network structure. The input required by this neural network is a 2D-matrix containing Fasta sequences of 50 kb length. Inpactor2_Detect is composed by two main sections. First, the data is processed by three convolutional layers of 32, 64 and 128 filters. Each convolution was followed by a Max Pooling layer of different sizes and strides. The last layer’ output was used as input for two fully connected layers of 1000 and 500 neurons. Finally, the prediction was done by a one-neuron-layer through a sigmoid action function.


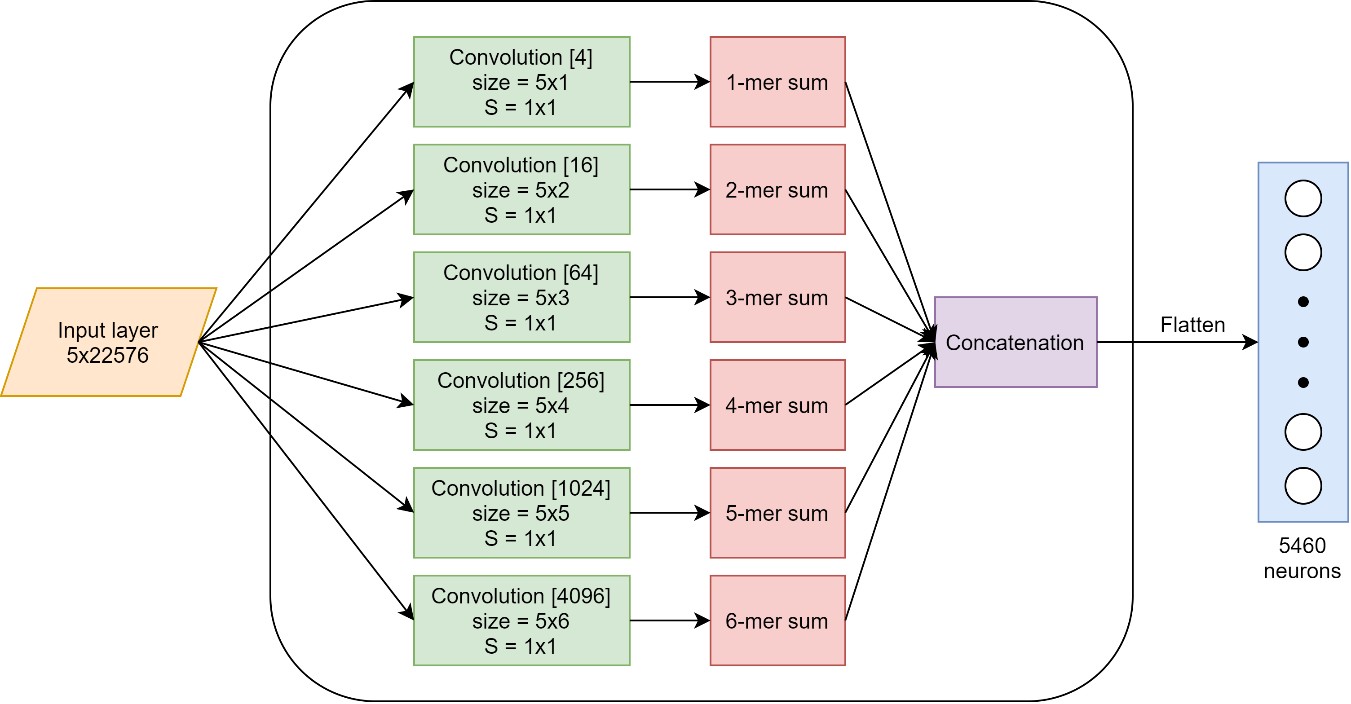


**Figure S3.** Inpactor2_*K*-mers convolutional neural network architecture for computing *k*-mer frequencies. It is composed by 6 convolutional layers, each one responsible to calculate the frequency of all possible *k*-mers using one value of *k*. Then, all the frequencies are summed and concatenated to obtain 5460 frequencies. The filters used in this network are non-trainable.


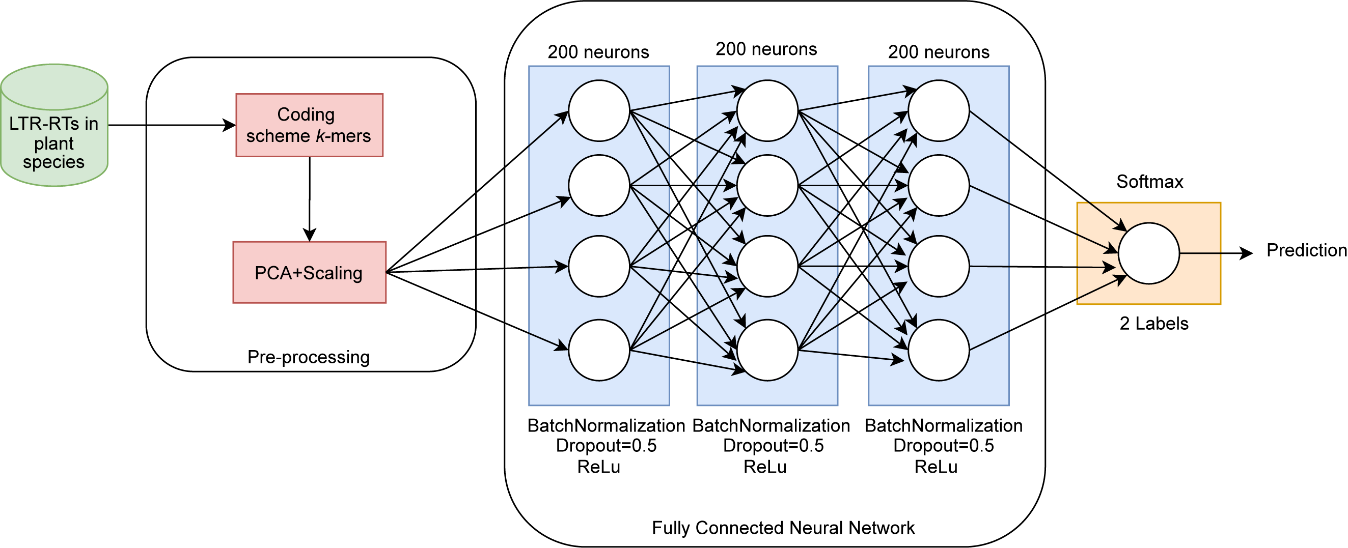


**Figure S4.** Inpactor2_Filter architecture. This network uses *k*-mer frequencies as features and required the data be scaled and reduced by Principal Component Analysis. It is composed by three fully connected layers each one with 200 neurons. A drop-out of 0.5 was used after each layer and ReLu was employed as activation function.


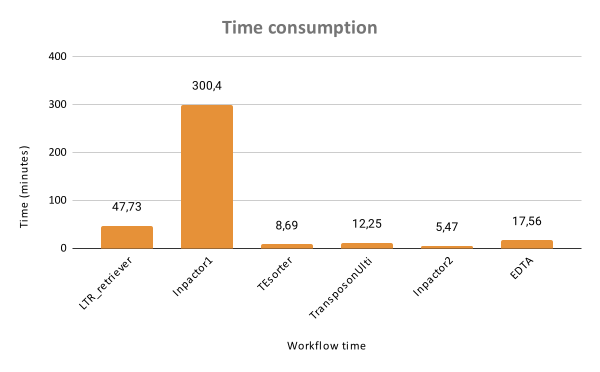


Figure S5. Execution times (Min) for each tool used based on the *O. sativa* genome as input.


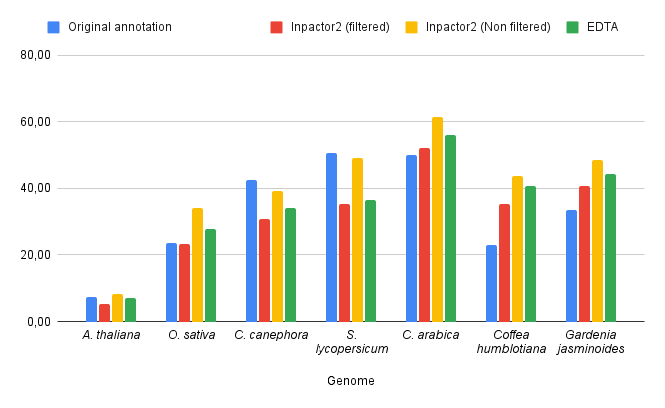


**Figure S6.** Proportion of LTR-RTs annotated from the original annotation, using Inpactor2 (with filtering and without it), and EDTA.


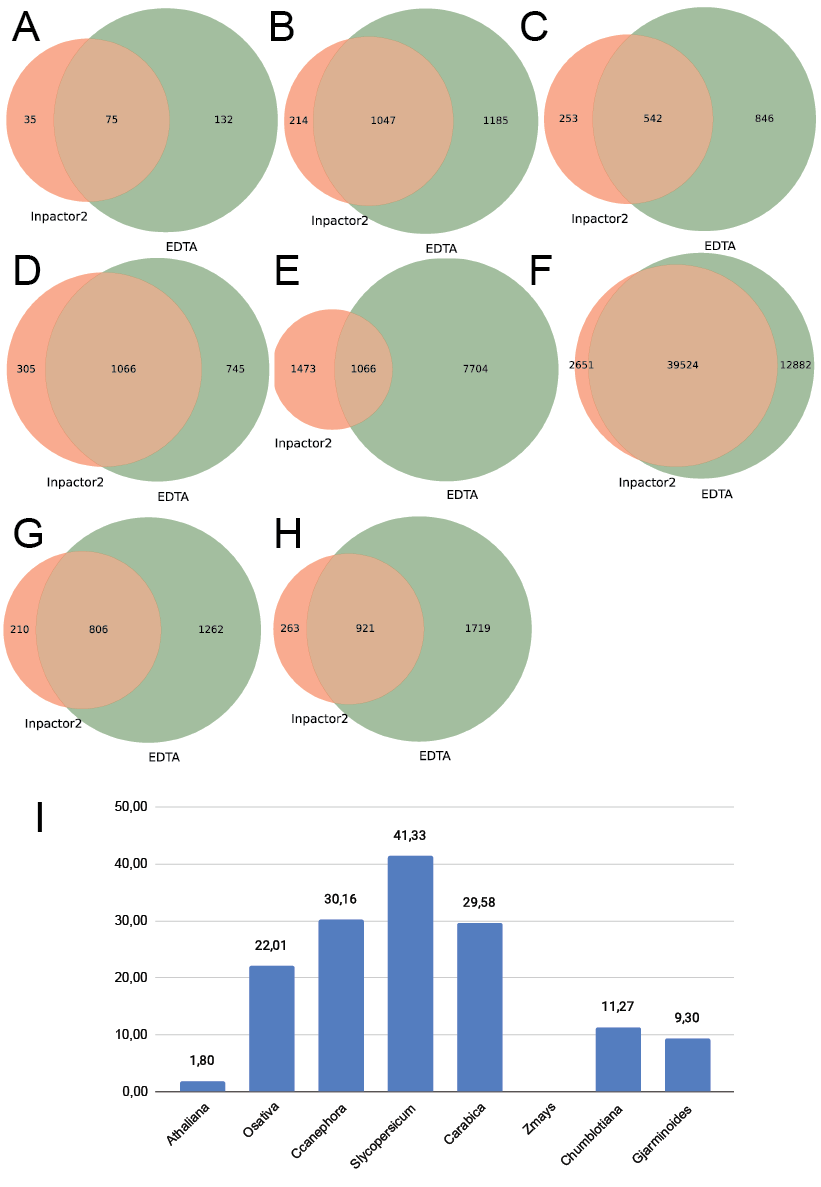


**Figure S7.** TE models shared between Inpactor2 and EDTA. Those sequences showed in the shared section of each diagram represent EDTA’s models that have a match with some Inpactor2’s model following the 80-80-80 rule (80% of identity, over the 80% of the sequence’s length and with a minimum of 80 bp) in A) *A. thaliana*, B) *O. sativa*, C) *C. canephora*, D) *S. lycopersicum*, E) *C. Arabica*, F) *Z. mays*, G) *C. humblotiana* and H) *G. jarminoides*. I) represents how many, in average, EDTA’s models satisfied the 80-80-80 law with Inpactor2’s models.


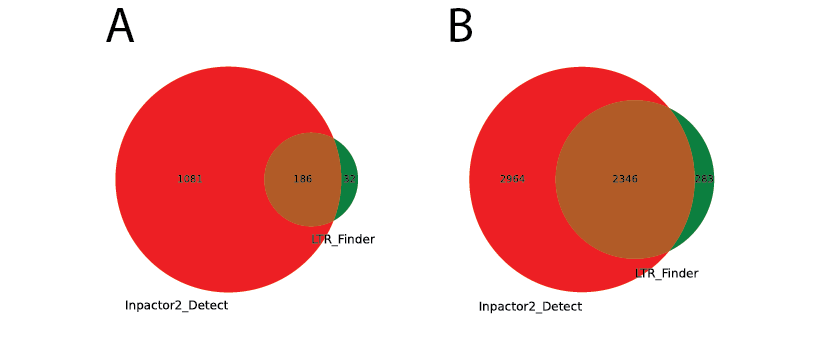


**Figure S8.** Venn diagram of genomic sections detected as containing LTR-RTs by the strategies used in the Hybrid approach in A) *A. thaliana* and B) *O. sativa*. Total number of sections can be viewed in Table S3.

| Metrics | Inpactor2_Detect | Inpactor2_Filter | Inpactor2_Class |
| --- | --- | --- | --- |
| Precision | 0.9712 | 0.9140 | 0.9869 |
| F1-score | 0.9711 | 0.9121 | 0.9874 |
| Recall | 0.9712 | 0.9125 | 0.9879 |
| Accuracy | 0.9712 | 0.9125 | 0.9879 |
| Area under ROC curve (AUC) | 0.9731 | 0.9632 | 0.9630 |
| Area under the Precision Recall | 0.9615 | 0.9660 | 0.9660 |

**Table S1**. Result metrics of Inpactor2_Detec, Inpactor2_Filter and Inpactor2_Class using the test dataset.

| Count method | Runtime (Sec) | Max memory (Gb) | Architecture |
| --- | --- | --- | --- |
| Inpactor2_K-mers (BS=1) | 8119,5 | 9,18 | GPU |
| Inpactor2_K-mers (BS=2) | 5737,9 | 9,17 | GPU |
| Inpactor2_K-mers (BS=128) | 2163,4 | 9,17 | GPU |
| Conventional | 9659,1 | 2,74 | CPU |

**Table S2**. Runtime and computational resources used by Inpactor2_K-mers and the conventional method based on CPU. InpactorDB (non-redundant version) sequences were used in these tests (more than 67 thousand). The execution time is the total time expended by each method to count all the possible *k*-mers in the entire dataset using 1<=*k*<=6. BS is batch size.

| Software | sensibility | specificity | accuracy | Precision | FDR | F1-Score |
| --- | --- | --- | --- | --- | --- | --- |
| LTR_FINDER-LTR_retriever | **0,96** | 0,94 | 0,94 | 0,83 | 0,17 | 0,89 |
| FASTA-Inpactor1 | 0,95 | 0,89 | 0,91 | 0,73 | 0,27 | 0,83 |
| LTR_FINDER-TEsorter | 0,88 | 0,94 | 0,92 | 0,82 | 0,18 | 0,85 |
| LTR_FINDER-TransposonUltimate | 0,92 | 0,92 | 0,92 | 0,78 | 0,22 | 0,84 |
| EDTA | 0,64 | **0,99** | 0,90 | **0,98** | **0,02** | 0,77 |
| Inpactor2 | 0,92 | 0,98 | **0,96** | 0,93 | 0,07 | **0,92** |

**Table S3**. Metrics achieved by each software using the *O. sativa* genome as input.

| Genomes | EDTA (sec) | Inpactor2 (sec) | Speed up |
| --- | --- | --- | --- |
| *A. thaliana* | 331.66 | 179.60 | 1.85 |
| *O. sativa* | 1053.74 | 328.54 | 3.21 |
| *C. canephora* | 838.62 | 345.12 | 2.43 |
| *S. lycopersicum* | 1310.37 | 855.31 | 1.53 |
| *C. arabica* | 7587.51 | 1568.59 | 4.84 |
| *Z. mays* | 74,932.76 | 10,512.24 | 7.13 |

**Table S4**. Timing consumption of EDTA and Inpactor2 using six plant genomes. Execution times are shown in seconds.

| Genomes | Inpactor2_Detect only | LTR_ finder only | Both positive | Both negative | Total |
| --- | --- | --- | --- | --- | --- |
| *A. thaliana* | 1081 | 32 | 186 | 1098 | 2397 |
| *O. sativa* | 2964 | 283 | 2346 | 1935 | 7528 |

**Table S5**. Comparison between the strategies used in the Inpactor2 hybrid approach. The column "Inpactor2_Detect only" shows how many genomic sections were predicted as containing LTR-RT by Inpactor2_Detect but missed by LTR_FINDER, while "LTR_FINDER only" shows the number of predictions made by LTR_FINDER, but missing in Inpactor2_Detect. The "Both positive" column shows the shared predictions of LTR-RT-containing sections, and "Both negative" shows the number of sections that were predicted as not containing LTR-RT by both strategies.

| Rice LTR-RT | Accession | Length (bp) | Reference | BLASTn vs Inpactor | E-value | %identity between reference and Inpactor |
| --- | --- | --- | --- | --- | --- | --- |
| DAGUL, Gypsy | AF537366.1 | 13425 | Vitte et al., 2007 | NC_029266.1_26646323_26659854#LTR/RLG/TEKAY-DEL | 0.0 | 89.9% |
| Hopi, Gypsy | AF537364.1 | 12892 | Vitte et al., 2007 | NC_029256.1_9531583_9544426#LTR/RLG/TAT | 0.0 | 100% |
| Houba/Tos5/Osr13, Copia | AF537365.1 | 6437 | Vitte et al., 2007 | NC_029265.1_19655030_19661451#LTR/RLC/TAR-TORK | 0.0 | 100% |
| osr42, Gypsy | AF458768.1 | 5605 | Vitte et al., 2007 | NC_029259.1_7930221_7935825#LTR/RLG/REINA | 0.0 | 98.2% |

**Table S6**. Well studied LTR retrotransposons presented in the *O. sativa* genome and the comparison with those found by Inpactor2.
